# Pituitary hCG production and cerebral tuberculosis mimicking disease progression during chemotherapy for an advanced ovarian germ cell tumour

**DOI:** 10.1186/1471-2407-10-338

**Published:** 2010-06-29

**Authors:** Serena Rakha, Clare Bayliss, Frances Sanderson, Richard Smith, Michael Seckl, Philip Savage

**Affiliations:** 1 Department of Medical Oncology Imperial College Healthcare NHS Trust Charing Cross Hospital London, UK; 2 Department of Medicine Imperial College Healthcare NHS Trust Charing Cross Hospital London, UK; 3 Department of Obstetrics and Gynaecology Imperial College Healthcare NHS Trust Queen Charlotte's Hospital London, UK

## Abstract

**Background:**

Ovarian germ cell tumours (OGCT) are rare but are usually curable with chemotherapy, even when presenting with advanced disease. The majority of OGCT produce the tumour markers, hCG and/or AFP which can be helpful in the diagnosis and monitoring the response to treatment.

**Case Presentation:**

In this case of a 36 year old woman, the elevated hCG level at presentation was helpful in making a clinical diagnosis of OGCT in a patient too unwell to permit a tissue diagnosis.

Cisplatin based combination chemotherapy produced an initial normalisation of the hCG level, but later in treatment the patient developed new cerebral lesions and a rising serum hCG suggestive of disease progression.

Further investigations suggested that the CNS lesions were cerebral TB and that the low levels of hCG elevations was likely to be pituitary in origin. Chemotherapy treatment was continued along with anti-tuberculous therapy and 24 months after successful completion of therapy the patient remains disease free.

**Conclusions:**

In the treatment of cancer patients it may be helpful to consider the potential non-malignant causes of new CNS lesions and that low hCG elevations may result from physiology rather than pathology in selected cases.

## Background

The diagnosis of a malignant ovarian germ cell tumour is rare with less than 100 new cases per year in the UK. For patients with advanced disease treatment with cisplatin based chemotherapy is generally curative with the overall cure rates approaching 80% [1]. Elevated tumour marker levels can be helpful in establishing the diagnosis in patients with advanced germ cell tumours as approximately 85% of cases of OGCT make one or both of the tumour markers hCG and AFP, with a poorer prognosis for those producing both at presentation [1]. During chemotherapy treatment the rate of the fall of the tumour markers can be helpful in assessing response and a failure to fall appropriately or to rise on treatment is associated with a poor outcome [2].

Tuberculosis remains a major public health issue worldwide and is currently increasing in incidence in the developed world. The reactivation of previously dormant TB in patients treated with steroids [3] or with chemotherapy is well documented [4], however the co-existence of TB in a patient with a malignancy is relatively unusual in developed countries with estimated TB incidence rates of 1:500-1:1000 cancer patients and most clinicians will see few if any cases [5].

In addition to production in pregnancy, trophoblast tumours and ovarian germ cell tumours, the production of low levels of hCG from the pituitary gland in post menopausal women has recently been documented. This physiological production of hCG has previously lead to diagnostic confusion in patients who were presumed to have occult gestational tumours and went on to receive unwarranted treatment [6].

A similar situation of physiological pituitary hCG production can occur in women receiving treatment for hCG producing malignancies. As a result of the ovarian suppression induced initially by the high hCG levels and then by their chemotherapy, these women can develop low oestrogen levels and a pattern of elevated LH, FSH and occasionally hCG levels similar to that seen in post menopausal women.

## Case presentation

A 36 year old woman was admitted to her local hospital following a collapse at work, she had a 3 month history of dyspnoea, weight loss and abdominal swelling.

**Figure 1:**
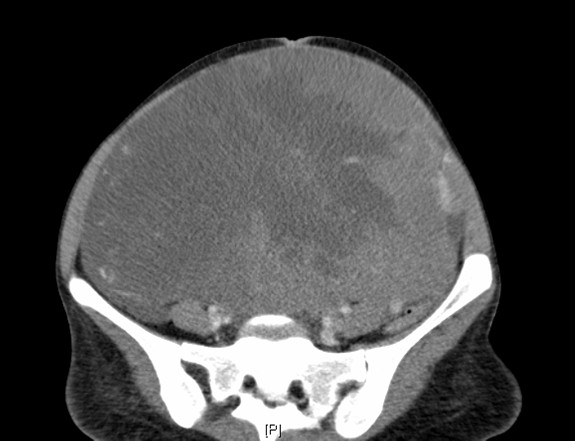
**CT scan of the abdomen on admission**. This scan demonstrates a large necrotic, partially cystic tumour occupying most of the abdomen. The CT scan also demonstrated marked paraaortic lymphadenopathy but no visceral or CNS metastases.

Clinical examination demonstrated a large mass occupying most of the abdomen and pelvis which was confirmed on CT scanning as shown in Fig 1. Additionally there were bilateral pleural effusions, but no other apparent pulmonary abnormalities. A CT scan of the brain showed no apparent abnormalities.

The routine haematology and biochemistry blood tests were unremarkable, however the serum hCG level was elevated at 1322 IU/L (nr 0-4), the CA-125 was also elevated at 1475 U/ml, whilst the serum AFP level was normal.

The clinical diagnosis made was of an advanced malignant ovarian germ cell tumour and shortly after transfer the patient's condition worsened with bowel obstruction and a pneumothorax in addition to the bilateral pleural effusions. This difficult clinical situation mandated rapid treatment and urgent chemotherapy was commenced with two days of etoposide (100 mg/m2) and cisplatin (20 mg/m2) using dexamethasone and ondansetron as the anti-emetics. The initiation of chemotherapy lead to a significant improvement in the clinical condition and the chemotherapy regimen was then changed to the POMB/ACE combination [7].

Following a brief stay in ITU for the treatment of hypotension and neutropaenic sepsis the patient was discharged home 1 month after admission to continue outpatient based chemotherapy with the hCG falling to normal 6 weeks after commencing treatment.

Two weeks after hospital discharge, the patient represented with a short history of impaired consciousness and pyrexia. Investigations demonstrated neutropaenia and hypokalaemia but with a normal CT scan of the brain and normal serum hCG levels.

**Figure 2:**
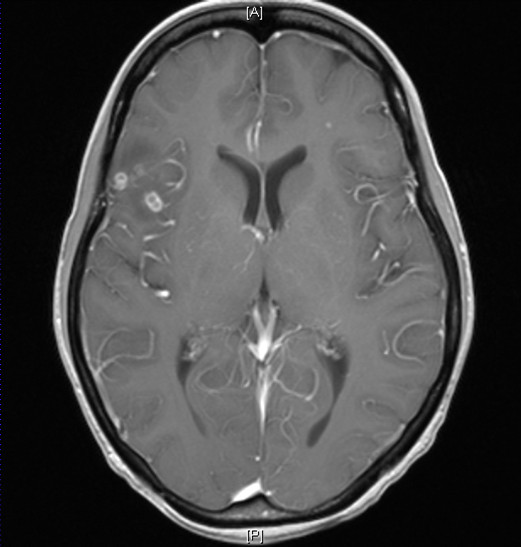
**MRI brain scan performed mid-treatment**. The MRI brain scan performed shortly after readmission with acute neurological symptoms, demonstrates a number of ring-enhancing lesions with surrounding oedema which were thought likely to be metastases.

At this point the diagnosis was unclear and the patient received empirical treatment for neutropaenic sepsis, viral encephalitis and tuberculosis. Two days later the patient's condition deteriorated further with the development of meningism and nystagmus and an MRI scan of the brain demonstrated oedema and a number of enhancing lesions reported as probable metastases as shown in Fig 2.

The empirical emergency treatment was continued with antibiotics, anti-virals, steroids and anti-TB therapy comprising Rifater, Pyridoxine, and Ethambutol and over the next 10 days the patient's condition improved gradually. The CSF sample taken at readmission was reported as positive for TB by both PCR and Elispot and culture positive growth was subsequently seen at 21 days.

Following the improvement from the acute neurological events, the POMB/ACE chemotherapy was recommenced but at this point the serum hCG level had become elevated with values ranging from 5-10 IU/L and this finding combined with an initial increase in the size and number of the CNS lesions on a repeat MRI again raised the concern that at least some of the lesions could be CNS metastases. However with the diagnosis of CNS TB confirmed and our previous experienced of transient hCG elevations in women treated with prolonged chemotherapy we elected to assume that all the lesions were infective in origin and persist with the chemotherapy treatment as planned.

**Figure 3:**
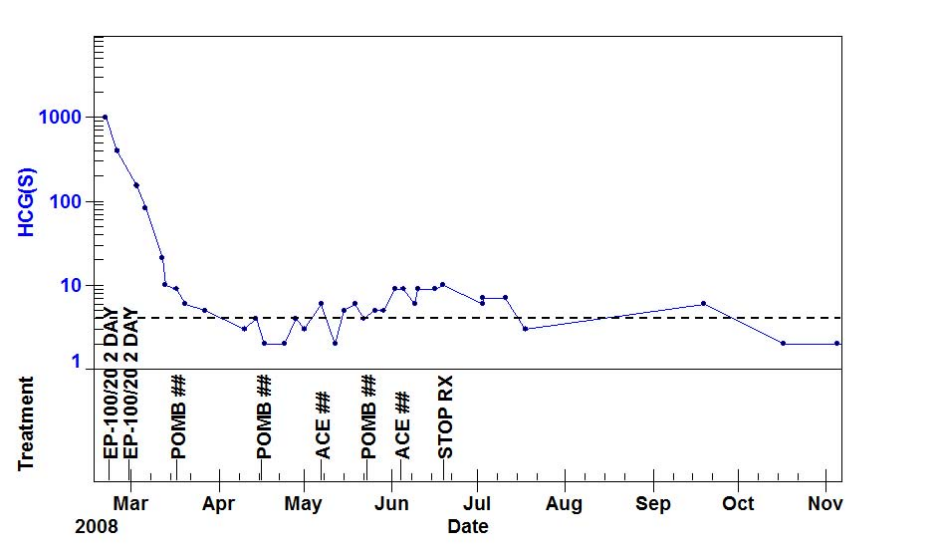
**hCG treatment graph**. This demonstrates the level of the serum hCG and the timing of chemotherapy treatment. After the initial elevation and fall to normal there was a 3 month low level hCG elevation. The solid line indicates the lower level of detection of the assay (1 IU/L) the dashed line the upper limit of the normal range (0-4 IU/L).

The POMB/ACE chemotherapy was concluded with a total of 16 weeks treatment, whilst the anti-TB therapy was continued for a full course of 6 months. During the remainder of chemotherapy and initially post treatment the hCG level remained elevated for approximately 3 months before returning to the normal range as shown in the treatment graph in Fig 3.

At the completion of chemotherapy, a laparotomy was performed, removing the residual tumour, the ovarian remnant, but leaving the uterus and contralateral ovary in place. The pathology from the resected tumour was reported as necrotic tissue in keeping with a germ cell origin but without any viable tumour.

**Figure 4:**
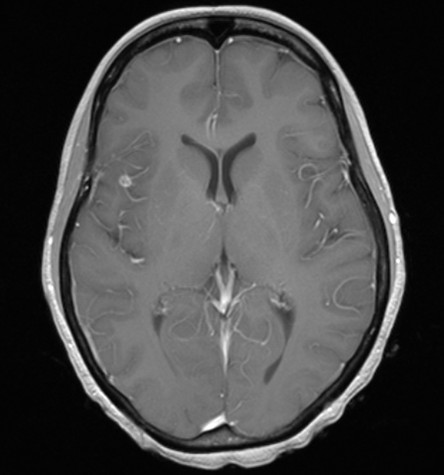
**MRI brain scan performed 9 months post chemotherapy**. The follow-up scan indicates that the previously noted oedema and the majority of the CNS lesions have resolved.

Post operatively the patient made an excellent returning to work 9 months after presentation with normal menstrual function returning 8 months after finishing chemotherapy. The follow up investigations have confirmed that the abdomen and pelvis remain free of disease and the CNS lesions have essentially resolved, as shown in Fig 4, and the hCG levels remain normal.

## Conclusions

Ovarian germ cell tumours characteristically occur in teenagers and young women and as a result of their rapid growth can present at advanced stages with clinically unwell patients.

From choice the diagnosis of OGCT should be confirmed histologically, either from oopherectomy in low stage disease or by biopsy in advanced disease. However in patients who are too unwell to tolerate an invasive procedure or the delay in confirming the diagnosis, then emergency treatment based on the likely clinical diagnosis can be warranted. In this case report the production of hCG by the tumour aided the clinical diagnosis pointing towards either a gestational tumour or an ovarian germ cell tumour as the most likely diagnoses. The relatively low level of hCG compared to the large bulk of the tumour favoured the diagnosis of an OGCT as the levels of expression can vary more widely in this diagnosis than in a gestational tumour and rarely reach very high levels.

Chemotherapy treatment leads to cure in the majority of patients with advanced OGCT, however in those with treatment resistance and particularly progression on therapy as generally displayed by rising markers or the development of new lesions the prognosis is poor. The group of GCT patients with the worst prognosis are those who develop CNS on treatment, for this group further chemotherapy treatment is rarely productive [8]. Fortunately in this case the developing CNS lesions were indicated as likely cerebral TB by PCR and Elispot analysis, and successfully treated with combination anti-tuberculous therapy. Alongside this the POMB/ACE chemotherapy was continued and has almost certainly cured the patient of the tumour.

A number of previous publications have highlighted the possibility of reactivation of TB occurring as a result of steroids or chemotherapy-induced immunosuppression [3-5], by far the most frequent sites of TB activity is in the lungs and it is extremely unusual for TB to present in the CNS without evidence of the disease elsewhere.

Generally hCG measurements give an accurate insight into the response of gestational and germ cell tumours to treatment with a very low incidence of false positive elevations. Recently it has been observed that modest elevations of hCG of pituitary origin can also occur of a part of normal physiology in post menopausal women in parallel with the high levels of LH and FSH produced [6]. A similar picture of presumed pituitary hCG production appears to take place in some women who have low oestrogen levels from inhibited ovarian function generally from prolonged chemotherapy and in this case report the patient had report a 6 month history of amenorrhea prior to starting chemotherapy that would have lead to low oestrogen levels at the start of treatment.

Whilst new CNS lesions and rising tumour marker levels would generally suggest metastasis and signify cancer progression, this case illustrates that there are differential diagnoses of both findings and very occasionally both will be due to non-malignant causes. In this unusual case chemotherapy was successfully continued, despite these concerning findings, and will probably have resulted in the cure of the patient.

## Consent

Written informed consent was obtained from the patient for publication of this case report and any accompanying images. A copy of the written consent is available for review by the Editor-in-Chief of this journal.

## Competing interests

The authors declare that they have no competing interests.

## Authors' contributions

SR and CB assembled the clinical data and wrote the paper. FS, RS, MS and PS were involved in the clinical care. PS is the corresponding author. All the authors have read and approved the final version of this paper.

## Pre-publication history

The pre-publication history for this paper can be accessed here:

http://www.biomedcentral.com/1471-2407/10/338/prepub
